# Statistical Power and Estimation of Incidence Rate Ratios Obtained from BED Incidence Testing for Evaluating HIV Interventions among Young People

**DOI:** 10.1371/journal.pone.0021149

**Published:** 2011-08-10

**Authors:** Bertran Auvert, Guy Séverin Mahiane, Pascale Lissouba, Thierry Moreau

**Affiliations:** 1 CESP INSERM-UVSQ UMRS-1018, Villejuif, France; 2 University of Versailles-Saint-Quentin, Versailles, France; 3 Assistance Publique-Hôpitaux de Paris, Hôpital Ambroise-Paré, Boulogne, France; 4 Institut Universitaire de France (IUF), Paris, France; 5 SACEMA, Stellenbosch University, Stellenbosh, South Africa; 6 CESP INSERM-Paris-11 UMRS-1018, Villejuif, France; Genentech Inc., United States of America

## Abstract

**Background:**

The objectives of this study were to determine the capacity of BED incidence testing to a) estimate the effect of a HIV prevention intervention and b) provide adequate statistical power, when used among young people from sub-Saharan African settings with high HIV incidence rates.

**Methods:**

Firstly, after having elaborated plausible scenarios based on empirical data and the characteristics of the BED HIV-1 Capture EIA (BED) assay, we conducted statistical calculations to determine the BED theoretical power and HIV incidence rate ratio (IRR) associated with an intervention when using BED incidence testing. Secondly, we simulated a cross-sectional study conducted in a population among whom an HIV intervention was rolled out. Simulated data were analyzed using a log-linear Poisson model to recalculate the IRR and its confidence interval, and estimate the BED practical power. Calculations were conducted with and without corrections for misclassifications.

**Results:**

Calculations showed that BED incidence testing can yield a BED theoretical power of 75% or more of the power that can be obtained in a classical cohort study conducted over a duration equal to the BED window period. Statistical analyses using simulated populations showed that the effect of a prevention intervention can be estimated with precision using classical statistical analysis of BED incidence testing data, even with an imprecise knowledge of the characteristics of the BED assay. The BED practical power was lower but of the same magnitude as the BED theoretical power.

**Conclusions:**

BED incidence testing can be applied to reasonably small samples to achieve good statistical power when used among young people to estimate IRR.

## Introduction

Since the first detuned enzyme immunoassay to detect recent HIV seroconversion was described in 1998 [1], there has been great interest in the application of laboratory methods to measure HIV incidence rates from cross-sectional samples [2]. Currently, the most widely used incidence assay is the BED HIV-1 Capture EIA (BED) assay [3]. HIV incidence estimation is increasingly being incorporated into HIV/AIDS surveillance activities in both resource-rich and developing countries [4]. However, in 2005, the UNAIDS Reference Group on Estimates, Modeling and Projections issued a cautionary statement about using BED to estimate HIV incidence rates and called for the development of additional laboratory and modeling methodologies [5].

The ability to reliably measure HIV incidence rate ratios (IRR) using cross-sectional data has vast public health importance in HIV surveillance and in prevention studies. In HIV surveillance, it will facilitate the identification of high risk groups. In HIV prevention studies, it will allow for the assessment of the roll-out of current interventions, such as male circumcision, or future interventions, such as microbicides and antiretroviral treatment as prevention. Reliable measures of HIV incidence rates from cross-sectional studies would reduce the need to recruit and maintain large and costly longitudinal cohorts. However, two of the current challenges in using HIV incidence assays to characterize HIV incidence rates are a) knowledge of the BED window period (i.e. time between first infection and when the test can reliably detect that infection) and b) misclassifications. The main source of misclassifications is the number of HIV-infected persons falsely identified as recent seroconverters, which depends on the proportion of HIV-positive participants whose infection duration exceeds the BED window period.

To reduce the sample size needed to achieve adequate statistical power, it would make sense to use BED incidence testing among young people because they constitute a group with a) a high HIV incidence rate and b) low HIV prevalence, since initiation of sexual activity is relatively recent.

The objectives of this study were to determine the capacity of BED incidence testing to a) estimate the effect of a prevention intervention and b) provide adequate statistical power, when used among young people from sub-Saharan African settings with high HIV incidence rates. Numerical values were obtained from published data.

## Methods

The details of the following calculations are provided in the Text S1, in which some computations were performed using a symbolic calculator [6].

### A theoretical estimations of the effect and of the power

We considered a population of young people at time t = 0, aged a_1_ to a_2_, from an area where HIV is predominantly transmitted heterosexually. We called T the maximum duration from their onset of sexual activity, which occurred at age a_1_, so that T = a_2_-a_1_. We supposed that the age distribution was uniform. This population was divided into a control group of size N and an intervention group of size N/m. The intervention was delivered at some time V years before t = 0, independently of HIV status. We supposed that all participants were HIV-negative at age a_1_, and that they were only tested for HIV after the end of the intervention. We assumed that HIV testing allowed us to a) perfectly detect HIV-positive and HIV-negative individuals, and b) imperfectly evaluate those having seroconverted during the BED window period (W). We called “tested recent seroconverters” these latter individuals, in contrast with those HIV-positive and long-term seroconverters. Misclassifications were due to participants who were falsely identified as recent seroconverters and those falsely identified as long-term seroconverters. The HIV incidence rate (i) was reduced by the effect (x) of the intervention, which was the IRR between the two groups. Because of the usually low value of the product of i by T, we approximated the probability of HIV infection during any duration lower than T by the product of the HIV incidence rate by this duration.

We adopted the terminology introduced by McDougal et al. [7], who considered three time intervals. The first interval was before HIV testing and equal in duration to the BED window period W. The second interval was immediately before the first interval and equal in duration to W. The third interval was the period before the first and the second intervals. The specificities associated with the second and third intervals are called the short-term (ρ_1_), and the long-term (ρ_2_) specificities. The sensitivity is noted S_e_. To simplify calculations, we considered that T was at least twice as long as W, which is the case in practice. We assumed that all the parameters were independent, and thus constant with time and age. In particular, we did not assume a) any relationship between the sensitivity and the specificities, b) any relationship between age and the sensitivity and the specificities, as it has been verified for the long-term specificity [8]. Any relationship between the sensitivity and the specificities can be taken into account when using numerical values.

Under these hypotheses, the mean number of those tested recent seroconverters (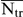) in the intervention group was given by the following formula:
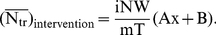
(1)In Formula 1, A and B are given by
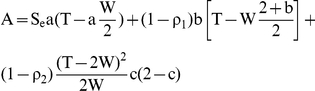


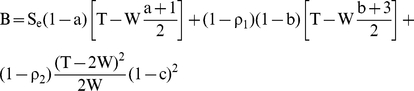
In these last formulae, a, b and c are fractions and depend on the duration of the intervention (V). They are given for V≤T by V = aW+bW+c(T-2W), and are equal to one for V≥T.

The number of individuals tested recent seroconverters in the control group was obtained from Formula 1 by replacing x by one.

The estimated effect of the intervention (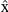) was calculated using the following formula: 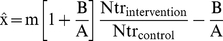
(2)As shown in the Text S1, the estimated effect calculated using Formula 2 is the maximum likelihood estimation of the effect.

The Text S1indicates the mathematical expression of the asymptotic (i.e. when N is large) 95% confidence interval (CI) of 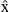.

The case of an intervention being delivered before or at the onset of sexual activity is discussed in section 5 of the Text S1. In this instance, the estimation of the intervention effect was equal to the ratio of the number of individuals tested recent seroconverters in the study groups, and did not depend on the characteristics of the BED incidence assay. We defined the BED theoretical power as the power obtained by statistical calculations. It appears that this BED theoretical power, and thus the calculation of the confidence interval, still depends on these characteristics.

In the Text S1, we also considered the case where the inferior limit of the age range was higher than the age at onset of sexual activity.

To avoid the hypotheses underlying the use of the delta method, and to circumvent the assumption that N is large, we ran simulations to compute the estimated effect of the intervention in function of N, m, i, W, T, V, ρ_1_, ρ_2_ and S_e_. We proceeded as follows: The distribution (D) of 10,000 values of the intervention effect was obtained by sampling the number of individuals tested recent seroconverters in each group from a binomial law, characterized by a number of trials equal to N (for the control group) or N/m (for the intervention group) and a probability equal to 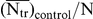 (for the control group) or 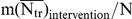 (for the intervention group). 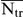 was calculated for each group as described above. The effect was calculated using Formula 2. In addition, the 0.025 to 0.975 percentile interval (Δ) of the effect distribution around a nominal value of one was obtained by generating another set of 10,000 values. The statistical power, which is called here the “BED theoretical power”, was given by the proportion of values of D outside the interval Δ.

This process was repeated by replacing the sensitivity and the specificities with one, to obtain the “cohort power”, which is the statistical power in the case of a classical cohort study. In such study, individuals, who are HIV-negative at recruitment, are followed-up for a duration of time equal to the BED window period and tested for HIV at the end of that duration, assuming no loss to follow-up. As shown in the Text S1, in some cases, the BED theoretical power can be slightly higher than the cohort power.

### B Practical estimation of the effect and of the BED power

In practice, the BED theoretical power estimation detailed above is not usable because empirical studies give individual data. Independently of the previous section, we examined how to estimate HIV IRR from empirical data using the following method: We considered an individual j aged g_j_ years-old and belonging to the intervention group. We considered that the duration of the intervention V_j_ varied between individuals. We defined T_j_ = g_j_-a_1_ and assumed that the intervention was delivered to that individual for 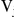 years, independently of his/her HIV status. We first calculated the theoretical probability of being tested recent seroconverter, for an HIV-positive individual having initiated sexual activity T_j_ years ago, with T_j_ lower than a given maximum value T_max_. Secondly, we simulated random samples of individuals belonging to the intervention and control groups, using these probabilities. These samples corresponded to what could be obtained when conducting a cross-sectional study. Lastly, we analyzed the simulated populations with classical statistical methods to estimate the value of the intervention effect, which was then used to simulate the data and assess the statistical power, called the “BED practical power” in this study. In this section, we use the same notations as in the previous paragraph.

#### Probabilities to be HIV positive and to be tested recent seroconverter

For an individual from the intervention group, the probability to be HIV-positive was given by the following formula:
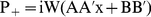
(3)The probability of being tested recent seroconverter when HIV-positive was given by the following formula:
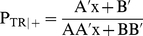
(4)In these formulae, A′ and B′ were calculated for five cases, depending on the relative values of T, W and V, and were independent of the HIV incidence rate. These cases are described in the Text S1. In the case of T≥2W, we obtained:
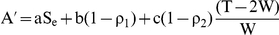


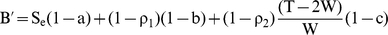
In each of the five cases, the values of AA′ and BB′ were obtained from A′ and B′ by replacing the specificities by zero and the sensitivity by one. For individuals in the control group, the probability to be HIV-positive and the probability to be tested recent seroconverter when HIV-positive were obtained by replacing x by one in Formulae 3 and 4.

#### Simulation of samples

We simulated an intervention group and a control group of the same size (N). The duration T since sexual debut was sampled from a uniform distribution between zero and the maximum T_max_. For individuals from the intervention group, the time V since the beginning of the intervention was sampled from a uniform distribution between a minimum (V_min_) and a maximum (V_max_). HIV status and, for those HIV-positive, results of the BED incidence testing (tested recent seroconverter or not) were randomly allocated according to Formulae 3 and 4. Each individual was then characterized by the three following variables: T, HIV status, and tested recent seroconverter (Yes or No) for those HIV-positive. In addition, the following general variables were used: N, i, x, W, Tmax, Vmin, Vmax, ρ_1_, ρ_2_ and V. This simulation process was repeated 10,000 times.

#### Estimation of the intervention effect and of the BED practical power

We recalculated the effect of the intervention, computed as an HIV IRR, from the simulated samples. We used a Poisson log-linear model which is equivalent to the exponential model [9], [10], [11], assuming that the instantaneous incidence is constant over time. This model was applied to all individuals from each simulation. This model required the knowledge of the probability k_1_ of being infected with HIV within the window period W for individuals tested HIV-positive and recent seroconverters, as well as the probability k_2_ of being infected with HIV within W for those tested HIV-positive and tested long-term seroconverters. As described in the Text S1, for intervention group participants, calculations gave the following expressions for k_1_ and k_2_.
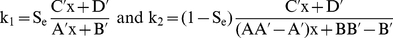
C′ and D′ were obtained from A′ and B′ by replacing the specificities and the sensitivity by one. AA′ and BB′ were obtained from A′ and B′ by replacing the specificities by zero and the sensitivity by one. The corresponding values of k_1_ and k_2_ for participants in the control group were obtained by replacing x by one. The values of k_1_ and k_2_ did not depend on the HIV incidence rate. To correct for an effect due to misclassifications (false recent seroconverters and false long-term seroconverters), weights of k_1_ were used for individuals tested recent seroconverters, weights of k_2_ were used for those tested long-term seroconverter, and weights of one were used for those tested HIV-negative. Because the weights depended on the effect of the intervention, Poisson log-linear regressions were repeated until a stable value for the intervention effect was reached. This process was initiated with a value of 0.5 for the first calculation of the weights. The duration of HIV exposure was introduced as an offset in the model. Each individual who remained HIV-negative was denoted as having a duration of exposure equal to the minimum value between W and T. Each individual who became HIV-positive was denoted as having a duration of exposure equal to half the minimum value between W and T. The estimated effect was calculated as the median effect generated from the 10,000 simulated samples. The statistical power needed to obtain a significant value for the HIV IRR was calculated as the fraction of the samples with a p-value lower than 0.05.

### C Numerical values

The conventional cut-off value for the BED assay is 0.80, corresponding to a BED window period W of about six months. However, an empirical study has shown that higher cut-off values of up to 1.89, corresponding to a W of about 15 months, can be used among young people [12]. We used values of W of six, nine, 12 and 15 months in this study. There are several approaches to establish the relationship between the cut-off value and W. One consists in considering a cohort of HIV-negative individuals followed up for a period of 2W, and then choosing a cut-off value such that, at the end of the 2W-period, the number of those who became HIV-positive during the second W period is equal to the number of those tested recent seroconverters. This is the method used in the publication quoted above, and what was applied in the present study. This method implies that the sensitivity is equal to the short-term specificity. Using other relationships between the cut-off value and W leads to other equations, such as sensitivity equals short-term specificity plus one minus long-term specificity [13]. The two methods give very close results because one minus the long-term specificity is in the range of 0.05 to 0.08.

Using empirical data from a previous study [12], we chose to use a linear relationship between the long-term specificity and W (in months), ρ_2_ = 1–0.0071W, and a constant value for the sensitivity, Se = 0.87. However, we also varied these numbers across a wide range of possible numerical values.

We selected baseline values for HIV incidence rates of 2.1% per year among young men and 5% per-year among young women, which are typical HIV incidence rates in high HIV incidence settings of sub-Saharan Africa. We chose an intervention reducing the HIV incidence rate by 60%, corresponding to an effect (IRR) of 0.4. Among men, these baseline data correspond to what was observed during the male circumcision trial conducted in Orange Farm (South Africa) [14].

### D Generation of results

#### BED theoretical power

We computed the theoretical power of the BED assay (the BED theoretical power) using the baseline scenarios. This BED theoretical power was also calculated as a fraction of the power obtained in a cohort study (called the cohort power). We assessed the effect of the baseline values on the BED theoretical power by varying a) the sample size of each group, b) the BED incidence assay window period W, c) the duration since sexual debut, d) the HIV incidence rate in the control group, e) the specificities and the sensitivity of the assay, f) the duration of the intervention and g) the effect of the intervention on HIV incidence rates.

#### Practical estimations

To assess the possibility of estimating the intervention effect, and to calculate the BED practical power when using a Poisson regression log-linear model on empirical data, we first simulated sets of control and intervention groups using the numerical values of the baseline scenarios. We then analyzed the simulated samples using the numerical values of the baseline scenario for the BED incidence assay characteristics (specificities, sensitivity and window period). This represented a theoretical situation where the characteristics of the BED assay are perfectly known, which is not the case in reality. To analyze the effect of these uncertainties, we analyzed the simulated population with characteristics for the assay different from those used to generate the samples. We also assessed the effect of not including offsets or weights when estimating the intervention effect and calculating the BED practical power. Not including weights corresponds to not correcting the data for misclassifications.

#### Programming

The program was written in R [15], and is available upon request from the corresponding author. In addition, we created a spreadsheet to calculate by simulation the BED theoretical power to detect an expected effect of an intervention when the following parameters are known: age at onset of sexual activity, age range, HIV incidence rate in the control group, expected effect of the intervention, duration of the intervention, and lastly the window period, sensitivity, and specificities of the BED assay. This spreadsheet is available upon request from the corresponding author.

### E Empirical example

We reanalysed the BED results obtained from data collected at the last follow-up visit of the Orange Farm male circumcision trial (ANRS-1265) [12], [14]. The research protocol for this trial was reviewed and approved by the University of the Witwatersrand Human Research Ethics Committee (Medical) on February 22nd, 2002 (protocol study no. M020104). In this trial, male participants, aged 18 to 24, were recruited from the general population of the township of Orange Farm (South Africa) and followed up for 21 months. The recruitment, randomization between intervention (male circumcision) and control groups, and follow-up were conducted independently of the participants HIV status. Among the 3274 participants recruited, 2949 were tested for HIV at the last follow-up visit (21-month visit). Among them, 2752 remained HIV-negative, 125 were HIV-positive at recruitment and 72 seroconverted during follow-up. Among the 197 HIV-positive samples at the last follow-up visit, 195 BED results were obtained. This dataset was analyzed using a mean reported age at first sexual intercourse of 16.7 years, assuming a) a constant HIV incidence rate and b) a linear increase of the HIV incidence rate from a value of zero at age 16.7. The intention-to-treat effect of male circumcision was calculated for various cut-off values and various values for the sensitivity and specificities indicated above. The estimated effects were qualitatively compared with the value obtained from classical survival analysis, which was 0.40 (95% CI: 0.24–0.68).

Lastly, we conducted a sensitivity analysis by varying the sensitivity and the specificities, in order to evaluate the impact of these changes on the estimated effect of the intervention.

## Results

### BED theoretical power

**Figure 1:**
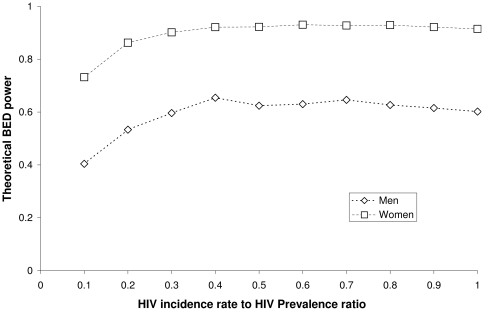
BED theoretical power as a function of the HIV incidence rate to HIV prevalence ratio. Results were obtained by simulations. The figure represents the BED theoretical power among young men and women as a function of the HIV incidence rate to HIV prevalence ratio for the baseline scenarios.

The baseline scenarios for young men (scenario 1) and young women (scenario 2) are detailed in Table 1. As shown in this table, the BED theoretical power, obtained by simulations to detect a significant effect, increases with increasing HIV incidence rate (scenario 2), BED window period W (scenarios 3, 4 and 5), sample size (scenario 6), and duration of the intervention (scenario 10). The power decreases with increasing time from sexual debut (scenarios 7 and 8), with decreasing intervention effect (scenario 9) and with decreasing specificities and sensitivity of the assay (scenarios 11 and 12). This table shows that the ratio between the BED theoretical power and the cohort power is always high, equal to 75% or more. Figure 1 shows the variation of the BED theoretical power with the ratio of the HIV incidence rate to HIV prevalence for the baseline scenarios. This power increases for ratios varying from 0.1 to 0.4, and then stabilizes.

**Table 1:** BED theoretical power needed to obtain a statistically significant HIV incidence rate ratio among young people when using BED incidence testing; results were obtained by simulations.

Scenario	1^b^	2^c^	3	4	5	6	7	8	9	10	11	12
**Data**												
Sample size in each group	1500	1500	1500	1500	1500	2000	1500	1500	1500	1500	1500	1500
BED Incidence assay window period (months)	6	6	9	12	15	6	6	6	6	6	6	6
Duration from sexual debut (years)	6	6	6	6	6	6	9	12	6	6	6	6
HIV incidence rate in the control group (% per year)	2.1	5.0	2.1	2.1	2.1	2.1	2.1	2.1	2.1	2.1	2.1	2.1
Short-term specificity	0.87	0.87	0.87	0.87	0.87	0.87	0.87	0.87	0.87	0.87	0.80	0.75
Long-term specificity	0.96	0.96	0.94	0.91	0.89	0.96	0.96	0.96	0.96	0.96	0.90	0.85
Sensitivity	0.87	0.87	0.87	0.87	0.87	0.87	0.87	0.87	0.87	0.87	0.80	0.75
Duration of the intervention (years)	2	2	2	2	2	2	2	2	2	4	2	2
Effect of the intervention^a^	0.4	0.4	0.4	0.4	0.4	0.4	0.4	0.4	0.5	0.4	0.4	0.4
**Results**												
BED theoretical power	0.62	0.91	0.73	0.77	0.85	0.72	0.53	0.50	0.45	0.70	0.54	0.53
Cohort power	0.66	0.93	0.80	0.88	0.94	0.75	0.67	0.67	0.47	0.66	0.65	0.66
BED theoretical power/Cohort power (%)	94	97	91	87	90	95	80	75	95	1.07	83	81
Incidence rate/Prevalence in the control group (per year)	0.33	0.33	0.33	0.33	0.33	0.33	0.22	0.17	0.33	0.33	0.33	0.33

a Factor by which the incidence rate is multiplied in the intervention group in comparison with the control group.

b Baseline scenario for young men.

c Baseline scenario for young women.

To obtain a power of 80%, with a BED window period of one year and with the other values being those of scenario 1, the sample size of each group should to be 670 for young women, assuming an HIV incidence rate of 5% per year, and 1,650 for young men, assuming an HIV incidence rate of 2.1% per year.

### Practical estimations

Table 2 presents the results obtained when estimating the intervention effect on simulated data using a log-linear Poisson model. It shows that this estimation is always relatively close to the real value. It also indicates that the cases where estimations are the poorest are when a) the characteristics of the assay are not known with precision (simulations 6 and 9) and b) the Poisson model is not weighted (simulations 3 to 5).

**Table 2:** HIV incidence rate ratio and BED practical power when using log-linear Poisson regression to analyze simulated samples of young people.

Simulation number	1	2	3	4	5	6	7	8	9	10	11
**Scenario # used to generate the sample**	1	1	1	1	1	1	1	2	2	2	12
**Values used to analyze the sample**											
Use of offset	Yes	No	Yes	Yes	Yes	Yes	Yes	Yes	Yes	Yes	Yes
Weights for tested recent^a^	k_1_	k_1_	1	k_1_	1	k_1_	k_1_	k_1_	k_1_	k_1_	k_1_
Weights for tested long-term^b^	k_2_	k_2_	k_2_	0	0	k_2_	k_2_	k_2_	k_2_	k_2_	k_2_
BED Incidence assay window period (months)	6	6	6	6	6	6	8	6	6	8	6
Short-term specificity	0.87	0.87	0.87	0.87	NA	0.80	0.87	0.87	0.80	0.87	0.75
Long-term specificity	0.96	0.96	0.96	0.96	NA	0.90	0.96	0.96	0.90	0.96	0.85
Sensitivity	0.87	0.87	0.87	0.87	NA	0.80	0.87	0.87	0.80	0.87	0.75
**Results**											
Number of tested recent											
Intervention	8	8	8	8	8	8	8	19	19	19	13
Control	17	17	17	17	17	17	17	42	42	41	24
Effect of the intervention^c^	0.39	0.39	0.44	0.39	0.44	0.35	0.40	0.37	0.34	0.39	0.38
95% CI: inferior limit	0.15	0.15	0.20	0.14	0.19	0.12	0.17	0.20	0.17	0.22	0.15
95% CI: superior limit	1.01	1.00	0.97	1.09	1.02	1.01	0.98	0.69	0.67	0.69	1.00
HIV incidence rate (% per year)	2.2		2.9	1.9	2.5	2.0	1.9	5.8	5.1	4.9	2.2
95% CI: inferior limit	1.3	NC	1.8	1.1	1.6	1.1	1.2	4.2	3.6	3.6	1.3
95% CI: superior limit	3.7		4.5	3.3	4.1	3.4	3.0	8.1	7.3	6.7	3.7
BED practical power	0.49	0.50	0.52	0.41	0.47	0.49	0.52	0.92	0.93	0.93	0.50
Average weights for tested recent^a^:											
Intervention	0.66	0.66	1	0.65	1	0.46	0.72	0.65	0.46	0.72	0.33
Control	0.75	0.75	1	0.75	1	0.58	0.79	0.75	0.58	0.79	0.46
Average weights for tested long-term^b^:											
Intervention	0.012	0.013	0.014	0	0	0.021	0.020	0.013	0.021	0.021	0.029
Control	0.024	0.024	0.024	0	0	0.039	0.036	0.025	0.039	0.038	0.052
BED practical/BED theoretical power^d^ (%)	0.79	0.81	0.84	0.66	0.76	0.79	0.84	1.01	1.02	1.02	0.94

NC = not calculable NA = Not applicable CI = confidence interval.

a b Weights k_1_ and k_2_ introduced in the Poisson log-linear model for each group a among HIV-positive tested recent seroconverters and b among HIV-positive tested long-term seroconverters.

c Factor by which the incidence rate is multiplied in the intervention group in comparison with the control group.

d The BED theoretical power was obtained from Table 1 (0.62 for simulations 1 thru 7, 0.91 for simulations 8 thru 10, and 0.53 for simulation 11).

In all other cases, this estimation ranged from 0.37 to 0.39 for a real value of 0.40. Table 2 also shows that the BED practical power was about 3/4 of the BED theoretical power for men and was about the same as the BED theoretical power for women. The average weights k_1_ ranged from 0.44 to 0.83 among the intervention group and from 0.61 to 0.93 among the control group.

To obtain a BED practical power equal to the cohort power indicated in Table 1 for the baseline scenarios (0.66 and 0.93), the sample size of each group had to be increased from 1,500 to 2,100 for men, and from 1,500 to 1,650 for women.

To obtain a BED practical power of 0.80 for the baseline scenarios, with a BED window period W of one year and corresponding values for the specificities and sensitivity, the sample size of each group was calculated to be 1,540 for men and 600 for women.

The calculations in the Text S1demonstrate that, in order to keep the weights k1, which correct for misclassifications, reasonably close to 1 (i.e., between 0.5 and 1), a) the time period from the onset of sexual debut to the upper age limit should be lower than 10 to 11 years and b) in the control group, the HIV incidence rate to HIV prevalence ratio should be higher than 0.09 to 0.10 year^−1^.

We generated datasets using the baseline scenarios but with a HIV incidence rate linearly increasing from age a_1_ to a_2_. The slope was selected in order to obtain the same number of HIV-positive cases in the control group when the HIV incidence rate was constant. We then analysed these datasets assuming a) a HIV incidence rate constant with age and b) a HIV incidence rate linearly increasing, as in the simulated data. In the first case, we obtained an effect of 0.33 (95% CI: 0.13–0.83) and 0.31 (95%CI: 0.17–0.57). In the second case, the values were 0.39 (95%CI: 0.18–0.85) and 0.37 (95%CI: 0.22–0.62).

### Empirical example

**Figure 2:**
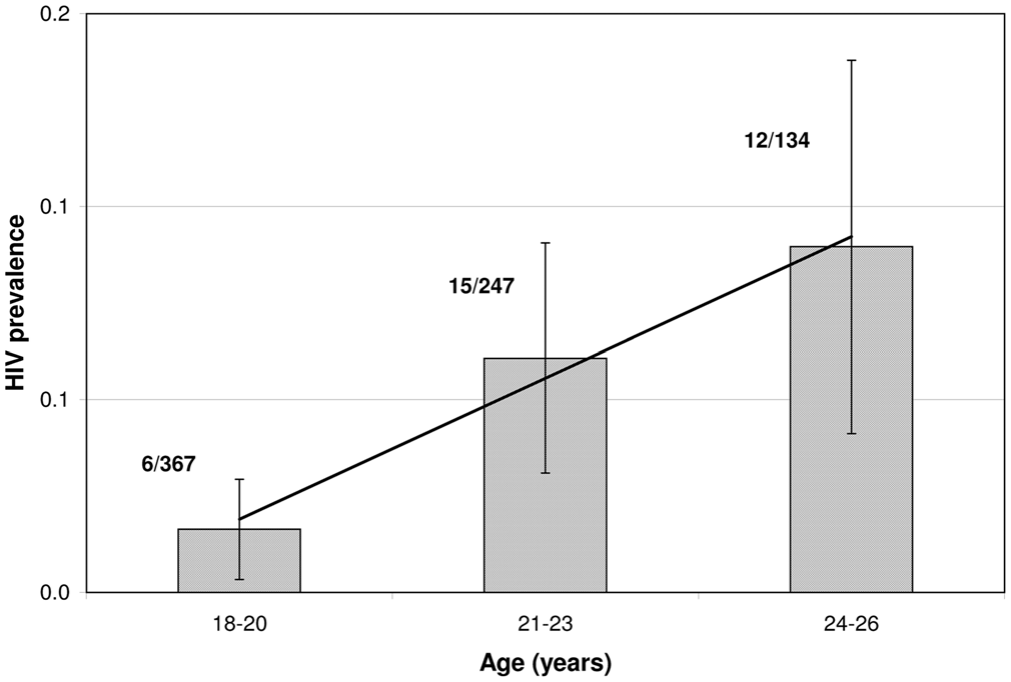
HIV prevalence by age obtained among uncircumcised men of the Orange Farm Community. This figure represents the HIV prevalence by age, with 95% confidence intervals, obtained among a random sample of uncircumcised men of the Orange Farm (South Africa) Community in 2007. A linear regression line has been added to the data.

The results are presented in Table 3. The values obtained for the effect of male circumcision on the HIV incidence rate, when assuming that this rate is constant, were in reasonably good agreement with the values obtained from classical survival analysis. The hypothesis of a constant HIV incidence rate is justified by the linear variation of HIV prevalence with age ranging from 18 to 26, as shown in Figure 2. HIV prevalence was zero for the age range 15 to 17. As shown in Table 3, the effect was statistically significant for all of the four BED cut-off values.

**Table 3:** Intention-to-treat effect of male circumcision estimated using BED incidence testing of blood samples obtained at the last follow-up visit of the Orange Farm male circumcision trial.

**Parameters**				
Cut-off	0.8	1.0	1.5	1.9
BED Incidence assay window period (months)	6.5	8.1	12.1	15.4
Short-term specificity	0.87	0.87	0.87	0.87
Long-term specificity	0.96	0.94	0.91	0.89
Sensitivity	0.87	0.87	0.87	0.87
Shape of HIV incidence rate by age	Constant	Constant	Constant	Constant
Weights for tested recent^a^	k_1_	k_1_	k_1_	k_1_
Weights for tested long-term^b^	k_2_	k_2_	k_2_	k_2_
**Results**				
Effect of the intervention^c^	0.42	0.36	0.34	0.37
95% CI	0.19–0.93	0.17–0.78	0.17–0.65	0.21–0.64

CI = confidence interval.

a b Weights k_1_ and k_2_ introduced in the Poisson log-linear model for each group a among HIV-positive tested recent seroconverters and b among HIV-positive tested long-term seroconverters.

c Factor by which the incidence rate is multiplied in the intervention group in comparison with the control group.

When we did not correct for the sensitivity (i.e. S_e_ = 1), we obtained the following values for the effect of the intervention: 0.43, 0.38, 0.35 and 0.38, which are very close to the values reported in Table 3.

When we did not correct for the short-term specificity (i.e. ρ_1_ = 1), we obtained the values 0.41, 0.35, 0.33 and 0.39, which are also very close to the values reported in Table 3.

When we did not correct for the long-term specificity (i.e. ρ_2_ = 1), we obtained the values 0.52, 0.49, 0.48 and 0.50, which are, in this case, underestimations of the effect of the intervention.

When we did not correct for misclassifications (i.e. k_1_ = 1 and k_2_ = 0, or S_e_ = ρ_1_ = ρ_2_ = 1), we obtained 0.53 (95%CI: 0.29–0.97), 0.49 (95%CI: 0.28–0.87), 0.49 (95%CI: 0.30–0.80) and 0.52 (95%CI: 0.35–0.80), which are also, and not surprisingly, underestimations of the effect of the intervention with, however, a statistically significant effect.

When we decreased the BED window period by 20%, the impact of the intervention was reduced by 0.6% to 1.9%. When we decreased the sensitivity by 20%, the impact of the intervention was changed by −2.1% to 4.2%. When we decreased the short-term specificity by 20%, the impact of the intervention was changed by −27% to 35%. When we decreased the long-term specificity by 20%, the impact of the intervention was increased from 4.4% to 5.3%.

## Discussion

Using theoretical analyses and mathematical simulations based on realistic scenarios, we investigated the performance of the BED incidence assay when used among young people recruited for a cross-sectional survey from high HIV incidence rate areas of sub-Saharan Africa. We were able to demonstrate that BED incidence testing a) has the ability to measure with substantial precision the effect of an HIV intervention aiming to reduce the HIV incidence rate and b) can lead to a statistical power close to the power obtained in classical cohort studies conducted among samples of the same size as the cross sectional survey and in which HIV-negative people are followed-up over a period of time equal to the BED window period.

An illustration of the use of BED incidence testing to assess the effect of an intervention was provided in another study published by some of the co-authors [12]. The study demonstrated that the protective effect of male circumcision could have been calculated using only blood samples collected from participants at the last follow-up visit of the Orange Farm male circumcision trial. This was shown using BED window periods ranging from six to 15 months, despite the presence at baseline of HIV-positive individuals who were followed-up exactly as HIV-negative participants. In the present study, the baseline scenario was based on data from the Orange Farm trial, and it demonstrated an adequate power and a precise estimation of the intervention effect. This is the reason why it was possible to replicate the results of the Orange Farm trial. However, the present study has a wider scope and its findings can be applied to various situations where the population consists of young people among whom HIV is predominantly transmitted heterosexually.

Our results are explained by the fact that in sub-Saharan African settings with high HIV incidence rates and where transmission is predominantly heterosexual, young people have been exposed to HIV for a short duration because of their recent onset of sexual activity. Hence, the use of the BED incidence assay is optimized because the ratio of HIV incidence rate with HIV prevalence is high. This high ratio leads to a lower absolute number of individuals falsely identified as recent seroconverters, in comparison with a population with a lower ratio, such as populations with wider age ranges. Another interest in studying young people is that the fraction of HIV-positive individuals on antiretroviral drugs or having low CD4 counts is lower than among older age cohorts because HIV infection is on average more recent. It ensues that young HIV positive people are more likely to be true recent seroconverters, hence the proportion of corrections for misclassifications due to these factors are lower [16], [17].

We found that a Poisson log-linear model, which is a classical multivariate statistical technique, can be used to analyze individual data obtained with BED incidence testing, and estimate with precision the effect of an intervention aiming to reduce HIV incidence rates. Such estimation was obtained by correcting for misclassifications, although it appears that a precise knowledge of the characteristics of the BED HIV incidence assay (sensitivity, specificities and window period) is not critical. This finding should lead to a wider use of these assays in HIV prevention research conducted among young people of sub-Saharan African settings with high HIV incidence rates. Moreover, such method has the ability to take into account cofactors of HIV incidence which can be included in the Poisson log-linear model. These cofactors may be collected among, or reported by, participants when a blood sample is also obtained. As such, the heterogeneity among participants can be accounted for, as long as it is not hidden. In addition, it is also possible to take into account propensity score weights. This allows to control for the selection bias due to a non-randomized intervention, when the risk behaviour of intervention and control group participants is likely dissimilar. The two approaches can even be combined into the so-called double robust estimation [18].

By searching the literature, we found only one study having used classical statistical methods to analyze risk factors of recent HIV infections identified with BED incidence testing [19]. However, to analyze risk factors of recent HIV infections is not the same as to use BED incidence testing to estimate HIV IRR. As shown in our study, correcting the BED incidence assay results to calculate HIV IRR leads to a better estimation of the intervention effect. Our study provides a theoretical framework for this type of estimation.

Our calculations demonstrate that sample sizes needed to estimate HIV IRR among young people and achieve an adequate statistical power can remain reasonably small. This result should change the assumption that HIV incidence assays are not practical because they require very large samples. Large samples may be needed to provide a precise estimation of HIV incidence rates among the general population [20].

Our theoretical and practical calculations of the estimated intervention effect, statistical power and confidence interval are general, but make the main assumption that the HIV incidence rate is constant as a function of time and age, as it is customary in this type of study [21]. To obtain simplified formulae, it was also assumed that the product of the HIV incidence rate by the duration since age at sexual debut is relatively small (i.e. no more than 0.35). These assumptions are acceptable when studying people from a limited age range, for a limited period of time, and with a recent onset of sexual activity. This is why our study concentrated on young people, even though our results are more general, as discussed in section A6 of the Text S1, where we considered an age range with a inferior limit higher than the age at onset of sexual activity.

Another interest in using incidence assays among young people is the possibility to increase the assay window period by increasing the cut-off value. For the BED assay, the conventional cut-off value is 0.80. However a literature search showed that this value has no real scientific basis. The possibility, when analyzing data from young people, to use higher cut-off values of up to 1.89, corresponding to a BED window period of about 15 months, has already been demonstrated in a recent study [12] and is consistent with theoretical studies showing that BED estimates can reflect an HIV incidence rate more than two years in the past [17]. A systematic review of the BED incidence assay [22] found that they could produce accurate estimates of HIV incidence rates, but that they were very sensitive to methodological and parameter choices. In particular, it was recommended that locally validated calibration parameters be used to compute incidence rates and correct for misclassifications [22]. Accordingly, our results were obtained with numerical values for the BED window period, the cut-off values, the specificities and the sensitivity yielded from a study conducted in Orange Farm among young men [12]. However, the mathematical expressions of our findings are general. Their use requires knowledge of the BED window period to correct for misclassifications, as well as the sensitivity and the specificities of the assay. These characteristics depend on the cut-off value used as well as the setting where the data are obtained.

We compared results obtained during a classical cohort studies with those obtained using the BED assay, with a window period equal in duration to the duration of the cohort studies follow-up period. However, cohort studies may have follow-up periods lasting longer than the window period. To compensate, the size of the cross-sectional survey during which BED incidence data is collected would have to be increased.

Vaccine, microbicide, pre-exposure prophylaxis or other potential HIV prevention or intervention studies would benefit from precise comparison of short-term incidence rates through the estimation of IRR using cross-sectional surveys. This requires that not all participants receive the intervention, otherwise no comparison will be possible and no IRR could be calculated. Cross-sectional HIV IRR measures would reduce the need to recruit longitudinal cohorts which are costly, and may suffer from recruitment bias.

Estimating the effect of an intervention by assessing the IRR is important in HIV prevention research. This effect may be more difficult to assess using longitudinal cohorts than cross-sectional studies. The current male circumcision roll-out study in Orange Farm (ANRS-12126), in which free medicalised male circumcision is made available to the community [23], was established to evaluate the effect of male circumcision on the HIV incidence rate in real life circumstances. The results of our study show that this effect may well be assessed by conducting a post-intervention cross-sectional survey. One additional advantage is that cross-sectional respondents are more likely to be representative of the population. Such characteristics are difficult to obtain when recruiting a cohort. Another example is Project Accept (National Institute of Mental Health, HPTN 043) [24], a community-based randomized trial providing community mobilization, mobile HIV voluntary counseling and testing, and comprehensive post-test supportive services. Measuring baseline HIV prevalence among the participating communities might have diluted the effect of the intervention, therefore the trial outcomes will be assessed using HIV incidence testing, and comparing IRR between control and intervention communities.

In conclusion, BED incidence testing may be employed to assess the effect of prevention interventions conducted among young people using cross-sectional data.

## Supporting Information

Text S1 **Theoretical calculations.**
(DOC)
