# Supplementary material for: Statistical Power and Estimation of Incidence Rate Ratios Obtained from BED Incidence Testing for Evaluating HIV Interventions among Young People
Source: PLoS One. 2011 Aug 10;6(8):e21149. doi: 10.1371/journal.pone.0021149 (PMC3154202; doi:10.1371/journal.pone.0021149)
Supplement: Text S1 — Theoretical calculations. (DOC) [file pone.0021149.s001.doc]

**Theoretical calculations**

**A Theoretical estimations of the effect and of the power**

**A1 Derivation of Formula 1**

We considered a population of young people at time t=0, aged a_1_ to a_2_, from an area where HIV is predominantly transmitted heterosexually. Ages are real number in years. We called T the maximum duration from their onset of sexual activity, which occurred at age a_1_, so that T=a_2_-a_1_. We supposed that the age distribution was uniform. This population was divided into a control group of size N and an intervention group of size N/m. The intervention was delivered at some time V years before t=0, independently of HIV status. We supposed that all participants were HIV-negative at age a_1_, and that they were only tested for HIV after the end of the intervention. We assumed that HIV testing allowed us to a) perfectly detect HIV-positive and HIV-negative individuals, and b) imperfectly evaluate those having seroconverted during the BED incidence window period (W). We called "tested recent seroconverters" these latter individuals, in contrast with those HIV-positive and tested long-term seroconverters. Misclassifications were due to participants who were falsely identified as recent seroconverters and those falsely identified as long-term seroconverters. The HIV incidence rate (i) was reduced by the effect of the intervention (x), which was the IRR between the two groups. Because of the usually low value of the product of i by T (i.e., not higher than 0.35), we approximated the probability of HIV infection during any duration (t) lower than T, $1-\text{e}^{-\text{it}}$, by the product of HIV incidence by this duration (it).

We adopted the terminology introduced by McDougal et al. [1], who considered three time intervals. The first interval is before HIV testing and equal in duration to W. The second interval is immediately before the first interval and equal in duration to W. The third interval is the period before the second interval. We labelled Ia, Ib and Ic the numbers of new infections which occurred during the first, second and third intervals, respectively. The sum Ia+Ib+Ic is the number (N+) of those HIV-positive at t=0. We called Na, Nb and Nc the number of individuals tested recent seroconverters among those infected during each interval. The sum Na+Nb+Nc is the number (Ntr) of those tested recent seroconverters at time t=0. We called Nr the real number of recent infections. The specificities associated with the second and third intervals are called the short-term (ρ_1_) and long-term (ρ_2_) specificities. The sensitivity is noted S_e_. The specificities and sensitivity were assumed to be independent of age. To simplify calculations, we considered that the age range was at least twice as long as the BED incidence assay window period (T≥2W), which is generally the case in practice.

The effective part of an intervention with V>T is T, because none of the individuals are sexually active when t<-T. The duration V can be formulated as V=aW+bW+c(T-2W). In this latter formula, a, b and c are fractions and are equal to one for V≥T. Table S1 indicates that the values of a, b and c depend on the value of V.

The following system 1 links the parameters and the variables.

$\text{ρ}_{2}=1-\frac{\text{Nc}}{\text{Ic}}$; $\text{ρ}_{1}=1-\frac{\text{Nb}}{\text{Ib}}$; $\text{Se}=\frac{\text{Na}}{\text{Ia}}$; $\text{Ntr}=\text{Na}+\text{Nb}+\text{Nc}$; $\text{Npos}=\text{Ia}+\text{Ib}+\text{Ic}$; $\text{Nr}=\text{Ia}$

We considered only the intervention group because the case of the control group was deduced by replacing x with 1 when it appeared in the formulae obtained for the intervention group.

Figure S1 illustrates how Ia, Ib and Ic were calculated. This figure represents the individuals history, from time t=-T to t=0. In this figure, the gray areas are not continuous to simplify the calculations, whereas in reality they are, as indicated by the possible values of a, b and c given in Table S1.

The average number of infections during each of the three intervals is the product of the number of individuals (in person-years) at risk with the incidence rate during that interval. Because this incidence rate is zero for individuals aged less than a_1_, one can see from Figure S1 that these numbers correspond to the product of the incidence rate a) with the area of the lower trapezoids (or triangles) and b) with the number of individuals per unit of age, which is N/(mT).

In the case of $\text{Ia}$, the time period to consider is between $-\text{W}$ and zero. The area of the gray domain is:

$\text{α}_{0}=\text{aW}\left[\text{T}-\frac{\text{a}}{2}\text{W}\right]$ while the remaining area is $\text{α}_{1}=\left(1-\text{a}\right)\text{W}\left[\text{T}-\frac{\text{a}+1}{2}\text{W}\right]$

Thus, we obtained$\text{Ia}=\frac{\text{NiW}}{\text{mT}}\left(\text{a}\left[\text{T}-\frac{\text{a}}{2}\text{W}\right]x+\left(1-\text{a}\right)\left[\text{T}-\frac{\text{a}+1}{2}\text{W}\right]\right)$.

Similarly, we obtained for Ib and Ic:

$\text{Ib}=\frac{\text{Ni}}{\text{mT}}\left(\text{xβ}_{0}+\text{β}_{1}\right)$and $\text{Ic}=\frac{\text{Ni}}{\text{mT}}\left(\text{xγ}_{0}+\text{γ}_{1}\right)$

with $\text{β}_{0}=\text{bW}\left(\text{T}-\text{W}\frac{\text{b}+2}{2}\right)$,$\text{β}_{1}=\left(1-\text{b}\right)\text{W}\left(\text{T}-\text{W}\frac{\text{b}+3}{2}\right)$

$\text{γ}_{0}=\frac{\text{c}}{2}\left(2-\text{c}\right)\left(\text{T}-\text{2W}\right)^{2}$and $\text{γ}_{1}=\frac{\left(1-\text{c}\right)^{2}}{2}\left(\text{T}-\text{2W}\right)^{2}$

Using system1, we obtained $\text{Na}=\text{SeIa}$,$\text{Nb}=\left(1-\text{ρ}_{1}\right)\text{Ib}$, and $\text{Nc}=\left(1-\text{ρ}_{2}\right)\text{Ic}$.

Inserting these latter formulae into Ntr=Na+Nb+Nc

gave $\text{N}_{\text{tr}}=\frac{\text{Ni}}{\text{mT}}\left(\left(\text{Seα}_{0}+\left(1-\text{ρ}_{1}\right)\text{β}_{0}+\left(1-\text{ρ}_{2}\right)\text{γ}_{0}\right)\text{x}+\text{Seα}_{1}+\left(1-\text{ρ}_{1}\right)\text{β}_{1}+\left(1-\text{ρ}_{2}\right)\text{γ}_{1}\right)$

Then, inserting the expressions of $\text{α}_{0}$, $\text{α}_{1}$, $\text{β}_{0}$, $\text{β}_{1}$, $\text{γ}_{0}$and $\text{γ}_{1}$given in the above formula yielded Formula 1 of the paper, which gave the mean number of individuals tested recent seroconverters ($\overline{\text{N}_{\text{tr}}}$):

$\text{(}\overline{\text{N}_{\text{tr}}}\text{)}_{\text{intervention}}=\frac{\text{iNW}}{\text{mT}}\text{(Ax}+\text{B)}$ (Formula 1)

in which A and B are given by

$$\text{A}=\text{S}_{\text{e}}\text{a(T}-\text{a}\frac{\text{W}}{2}\text{)}+\text{(1}-\text{ρ}_{1}\text{)b}\left[\text{T}-\text{W}\frac{2+\text{b}}{2}\right]+\text{(1}-\text{ρ}_{2}\text{)}\frac{\text{(T}-\text{2W)}^{2}}{\text{2W}}\text{c(2}-\text{c)}$$

$$\text{B}=\text{S}_{\text{e}}\text{(1-a)}\left[\text{T-W}\frac{\text{a}+1}{2}\right]+\text{(1}-\text{ρ}_{1}\text{)(1}-\text{b)}\left[\text{T}-\text{W}\frac{\text{b}+3}{2}\right]+\text{(1}-\text{ρ}_{2}\text{)}\frac{\text{(T}-\text{2W)}^{2}}{\text{2W}}\text{(1-c)}^{2}$$

We then considered that after a median period S from HIV acquisition, individuals either died or received antiretroviral therapy (ARV). We also considered that individuals under ARV were all identified, for example by testing their blood. Because S is larger than 2W, only Ic had to be reduced for those individuals when S was smaller than T. The factor that had to be subtracted from Ic is given in Table S2 for participants of the intervention group. The corresponding values for the control group were obtained by replacing x by one.

It results that the new values of A and B are the following:

$$\text{A}=\text{S}_{\text{e}}\text{a(T}-\text{a}\frac{\text{W}}{2}\text{)}+\text{(1}-\text{ρ}_{1}\text{)b}\left[\text{T}-\text{W}\frac{2+\text{b}}{2}\right]+\text{(1}-\text{ρ}_{2}\text{)}\left[\frac{\text{(T}-\text{2W)}^{2}}{\text{2W}}\text{c(2}-\text{c)-ε}_{\text{s}}\frac{\left(\text{T-S}\right)^{2}}{\text{2W}}\text{s(2-s)}\right]$$

$\text{B}=\text{S}_{\text{e}}\text{(1-a)}\left[\text{T-W}\frac{\text{a}+1}{2}\right]+\text{(1}-\text{ρ}_{1}\text{)(1}-\text{b)}\left[\text{T}-\text{W}\frac{\text{b}+3}{2}\right]+\text{(1}-\text{ρ}_{2}\text{)}\left[\frac{\text{(T}-\text{2W)}^{2}}{\text{2W}}\text{(1-c)}^{2}-\text{ε}_{\text{s}}\frac{\left(\text{T-S}\right)^{2}}{\text{2W}}\text{(1-s)}^{2}\right]$With $\text{ε}_{\text{s}}$equal to zero when S≥T, and $\text{ε}_{\text{s}}$equal to one otherwise. In general, $\text{ε}_{\text{s}}$is zero for young people because in this population, T is generally smaller than S, which has a typical value of 10 years.

**A2 Derivation of Formula 2 and maximum likelihood estimation of the effect**

From Formula 1, we obtained: $\text{(}\overline{\text{N}_{\text{tr}}}\text{)}_{\text{control}}=\frac{\text{iNW}}{\text{T}}\text{(A}+\text{B)}$. Thus the estimated effect of the intervention ($\hat{\text{x}}$) could be calculated using the following formula: $\hat{\text{x}}=\text{m}\left[1+\frac{\text{B}}{\text{A}}\right]\frac{\text{Ntr}_{\text{intervention}}}{\text{Ntr}_{\text{control}}}-\frac{\text{B}}{\text{A}}$ (Formula 2)

Formula 2 gives the maximum likelihood estimator of x. In fact, assuming that Ntr in the intervention and control groups are following independent binomial laws,

$\text{Ntr}_{\text{intervention}}=\text{B(}\frac{\text{N}}{\text{m}},\frac{\text{iW}}{\text{T}}\text{(Ax}+\text{B)})$and $\text{Ntr}_{\text{control}}=\text{B(N,}\frac{\text{iW}}{\text{T}}\text{(A}+\text{B))}$

By calling $d=\frac{\text{NW}}{\text{T}}$, the likelihood function is

$$\text{L}=\left[\text{i}\frac{\text{d}}{\text{m}}\text{(Ax}+\text{B)}\right]^{\text{Ntr}_{\text{intervention}}}\left[\text{id(A}+\text{B)}\right]^{\text{Ntr}_{\text{control}}}\left[1-\text{i}\frac{\text{d}}{\text{m}}\text{(Ax}+\text{B)}\right]^{\frac{\text{N}}{\text{m}}-\text{Ntr}_{\text{intervention}}}\left[1-\text{id(A}+\text{B)}\right]^{\text{N}-\text{Ntr}_{\text{control}}}$$

The maximum likelihood estimator of x is obtained by solving the system

$\frac{\partial\text{L}}{\partial\text{i}}=0$ and $\frac{\partial\text{L}}{\partial\text{x}}=0$ , which gives $\hat{\text{i}}=\frac{\text{T}}{\text{W}}\frac{\text{Ntr}_{\text{control}}}{\text{N(A}+\text{B)}}$ and Formula 2.

When N is large, $\hat{x}\approx x$

**A3 95% confidence interval of the maximum likelihood estimation of the effect**

Using the delta method for **,** which can be approximated by a normal distribution, we obtained the following 95% confidence interval for $\hat{\text{x}}$:

with$\sigma^{2}=\frac{1}{\text{N}}\left(\frac{\text{mT}}{\text{Wi}\left(\text{Ax}+\text{B}\right)}+\frac{\text{T}}{\text{Wi}\left(\text{A}+\text{B}\right)}-\left(1+\text{m}\right)\right)$

**A4 Comparison of the cohort power with the BED theoretical power**

When using the BED incidence assay, the estimation of the intervention effect ($\hat{\text{x}}$) is obtained from Formula 2, in which, as seen above, Ntr in the intervention and Ntr in the control groups are independent and follow binomial laws:

$\text{Ntr}_{\text{intervention}}=\text{B(}\frac{\text{N}}{\text{m}}\text{,}\frac{\text{iW}}{\text{T}}\text{(Ax}+\text{B))}$and $\text{Ntr}_{\text{control}}=\text{B(N,}\frac{\text{iW}}{\text{T}}\text{(A}+\text{B))}$. The case of a classical cohort of two groups of N people followed up during W years can be derived from this latter case by replacing the specificities and the sensitivity by one.

The BED theoretical power is defined as the statistical power obtained when estimating the intervention effect with the BED incidence assay. The cohort power is the statistical power obtained in the case of a classical cohort study of individuals, HIV-negative at recruitment, followed-up over a period of W years, and tested at the end of that period using the BED incidence assay, assuming no loss to follow-up.

In some cases, the BED theoretical power obtained when using the assay can be slightly higher than the power obtained in a cohort study. Let us consider the case when V≥ T. We have a=b=c=1. It results that B=0. Therefore, in this instance, the BED theoretical power is higher than the cohort power if the value of A is larger than the value of A obtained when the specificities and the sensitivity are replaced by one. For example, with N=1500, W=0.5 year, T=6 years, i=0.021 per year, $\text{ρ}_{1}$=0.87, $\text{ρ}_{2}$=0.96, Se=0.87, V=6 years and x=0.4, we obtained A=6.69 and a BED theoretical power of 0.73. With $\text{ρ}_{1}$=1, $\text{ρ}_{2}$=1 and Se=1, we obtained A=5.75 and a cohort power of 0.65.

**A5 Estimation of the effect and the BED theoretical power in the particular case of V≥T and S≥T**

This common case is defined when V and S are larger than or equal to T, and corresponds to an intervention being delivered to young people before the onset of sexual activity. In the case of male circumcision for example, it occurs when men are circumcised during childhood. In this instance, a, b, c are equal to one and $\text{ε}_{\text{s}}$= 0. Hence, B equals zero. It results from Formula 1 that the ratio of the number of individuals tested recent seroconverters in each group is equal to , that is : $\hat{\text{x}}=\text{m}\frac{\text{Ntr}_{\text{intervention}}}{\text{Ntr}_{\text{control}}}$

However, even in this case, the power calculation requires the estimation of A, which depends on the specificities, the sensitivity and the BED window period W, and which is given by the following formula:

$$\text{A}=\text{S}_{\text{e}}\text{(T}-\frac{\text{W}}{2}\text{)}+\text{(1}-\text{ρ}_{1}\text{)}\left[\text{T}-\frac{3\text{W}}{2}\right]+\text{(1}-\text{ρ}_{2}\text{)}\frac{\text{(T}-\text{2W)}^{2}}{\text{2W}}$$

If, as it is the case in practice, we assume that T is large in comparison with 2W and that Se is large in comparison with 1-, then A can be estimated by: $\text{A}=\text{S}_{\text{e}}\text{T}+\text{(1}-\text{ρ}_{2}\text{)}\frac{\text{T}^{2}}{\text{2W}}$. It results that the power depends predominantly on the age range, the BED window period, the long-term specificity and the sensitivity. It also follows that the confidence interval of $\hat{\text{x}}$ will largely depend on these same factors.

**A6 Estimation of the effect and the BED theoretical power in the particular case when the inferior age limit is higher than the age at onset of sexual activity**

In this section, we consider (1+1/m)N individuals with an age range from a_3_ to a_2_ with a_1_<a_3_<a_2_. We called T' the duration a_3_-a_1_. c becomes c' and is given in Table S1 with T replaced by T’.

We assumed that T and T' were larger than 2W. Calculations similar to those in section A1 show that $\text{(}\overline{\text{N}_{\text{tr}}}\text{)}_{\text{intervention}}=\frac{\text{iNW}}{\text{m(T-T')}}\text{(Ux}+\text{Q)}$ (Formula 1b), in which U and Q are given by the following formulae, with the functions A and B defined in section A1:

$\text{U}=\text{A( a, b, c, T, W, ρ}_{1}\text{, ρ}_{2}\text{, S}_{\text{e}}\text{)-A( a, b, c', T', W, ρ}_{1}\text{, ρ}_{2}\text{, S}_{\text{e}}\text{)}$ and

$$\text{Q}=\text{B( a, b, c, T, W, ρ}_{1}\text{, ρ}_{2}\text{, S}_{\text{e}}\text{)-B( a, b, c', T', W, ρ}_{1}\text{, ρ}_{2}\text{, S}_{\text{e}}\text{)}$$

The maximum likelihood estimator of x is $\hat{\text{x}}=\text{m}\left[1+\frac{\text{Q}}{\text{U}}\right]\frac{\text{Ntr}_{\text{intervention}}}{\text{Ntr}_{\text{control}}}-\frac{\text{Q}}{\text{U}}$ (Formula 2b)

When N is large, .

Using the delta method, we obtained the following 95% confidence interval for $\hat{\text{x}}$:

with$\sigma^{2}=\frac{1}{\text{N}}\left(\frac{\text{m(T-T')}}{\text{Wi}\left(\text{Ux}+\text{Q}\right)}+\frac{\text{T-T'}}{\text{Wi}\left(\text{U}+\text{Q}\right)}-\left(1+\text{m}\right)\right)$

**B Practical estimation of the effect and the power**

**B1 Derivation of Formula 4, k_1_ and k_2_**

Independently of the previous sections, we explored how to estimate HIV IRRs from empirical data using the following method: We considered an individual j aged g_j_ years-old and belonging to the intervention group. We considered that the duration of the intervention V varied between individuals. We defined T_j_=g_j_-a_1_ and assumed that the intervention was delivered to that individual for$\text{V}_{\text{j}}$years independently of his/her HIV status. From this point onwards, to simplify the writing, we will omit the subscript j when considering the variables T, g, V, a, b, c, A', B', AA', BB', C', D'. As in section A1, the intervention reduced the HIV incidence rate by a factor of $\text{x}$, such that the incidence rate was $\text{ix}$during the V years preceding the time $\text{t}=0$ at HIV testing.

The probability to be tested recent seroconverter was calculated by considering the history of the individual j and another time-age diagram. We found that this probability could be formulated as $\text{P}_{\text{TR}}=\text{iW(A}^{\text{'}}\text{x}+\text{B}^{\text{'}}\text{)}$with A' and B' given in Table S3.

We then considered that after a median period S following HIV acquisition, individuals either died or received ARV. We also considered that individuals under ARV were all identified, for example by testing their blood. Because S is larger than 2W, only Ic had to be reduced for those individuals when S was smaller than T. The factor that had to be subtracted from Ic is given in Table S4 for participants of the intervention group. The corresponding values for the control group were obtained by replacing x by one.

It results that the new values of A' and B' for T≥ 2W are the following:

$$\text{A'}=\text{aS}_{\text{e}}+\text{b(1}-\text{ρ}_{1}\text{)}+\text{(1}-\text{ρ}_{2}\text{)}\left[\text{c}\frac{\text{(T}-\text{2W)}}{\text{W}}-\text{ε}_{\text{s}}\text{s}\frac{\text{(T}-\text{S)}}{\text{W}}\right]$$

$$\text{B'}=\text{S}_{\text{e}}\text{(1-a)}+\text{(1}-\text{ρ}_{1}\text{)}(\text{1-b)}+\text{(1}-\text{ρ}_{2}\text{)}\left[\text{(1}-\text{c)}\frac{\text{(T}-\text{2W)}}{\text{W}}\text{-ε}_{\text{s}}\text{(1}-\text{s)}\frac{\text{(T}-\text{S)}}{\text{W}}\right]$$

With $\text{ε}_{\text{s}}$equal to zero for S≥T and one otherwise. In general, $\text{ε}_{\text{s}}$is zero for young people because in this population, T is generally smaller than S, which has a typical value of 10 years.

The duration of the intervention can be formulated as$\text{V}=\text{aW}+\text{bW}+\text{c}\left(\text{T}-\text{2W}\right)$. The values of a, b and c are given in Table S5. They depend on the relative values of T in comparison with W and 2W.

The probability to be HIV-positive can be written as $\text{P}_{+}=\text{iW(AA}^{\text{'}}\text{x}+\text{BB}^{\text{'}}\text{)}$, with AA' and BB' obtained from A' and B' by replacing the specificities by zero and the sensitivity by one. Because individuals tested recent seroconverters are among those who are HIV-positive, the probability of being tested recent seroconverter if HIV-positive was given by the following formula, which is Formula 4 of the paper:

$\text{P}_{\text{TR}\left|+\right.}=\frac{\text{A'x}+\text{B'}}{\text{AA'x}+\text{BB'}}$ (Formula 4)

When the HIV incidence rate, i, cannot be considered small, i.e. when an expression such as $1-e^{-it}$cannot be replaced by it, the exact values of A' and B' are given in Table S6.

**B2 Derivation of k_1_ and k_2_**

As seen above, the probability to be tested recent seroconverter was $\text{P}_{\text{TR}}=\text{iW(A}^{\text{'}}\text{x}+\text{B}^{\text{'}}\text{)}$. It results that the probability to be infected during the W years preceding t=0 can be formulated as $\text{P}_{\text{R}}=\text{iW(C}^{\text{'}}\text{x}+\text{D}^{\text{'}}\text{)}$, with C' and D' obtained from A' and B' by replacing the specificities and the sensitivity by one. Hence, the probability to be HIV-positive can be written as $\text{P}_{+}=\text{iW(AA}^{\text{'}}\text{x}+\text{BB}^{\text{'}}\text{)}$, with AA' and BB' obtained from A' and B' by replacing the specificities by zero and the sensitivity by one. Lastly, the probability to be tested recent seroconverter and being truly recently infected was $\text{P}_{\text{R} \text{and TR}}=\text{S}_{\text{e}}\text{iW(C}^{\text{'}}\text{x}+\text{D}^{\text{'}}\text{)}$. Hence k_1_, which is$\text{P}_{\text{R }\left| \text{TR}\right.}$, and k_2_, which is$\text{P}_{\text{R }\left| \text{not(TR}\right.\text{)}}$, are given by the following expressions:

$\text{k}_{1}=\text{S}_{\text{e}}\frac{\text{C'x}+\text{D'} }{\text{A'x}+\text{B'}}$ and $\text{k}_{2}=(1-\text{S}_{\text{e}})\frac{\text{C'x}+\text{D'} }{\text{(AA'-A')x}+\text{BB'-B'}}$

**B3 Calculation of k_1_ and k_2_ in the particular case of V≥T, S≥T and T≥2W**

This case corresponds to an intervention being delivered a) before the onset of sexual activity and b) among young individuals having started their sexual activity more than 2W years ago. In this case, a=b=c=1 and $\text{ε}_{\text{s}}$= 0. It results that $\text{A'}=\text{S}_{\text{e}}+\text{(1}-\text{ρ}_{1}\text{)}+\text{(1}-\text{ρ}_{2}\text{)}\frac{\text{(T}-\text{2W)}}{\text{W}}$, B'=0, C'=1, D'=0, $\text{AA'}=2+\frac{\text{(T}-\text{2W)}}{\text{W}}$ and BB'=0.

The expressions of k_1_ and k_2_ become

$\text{k}_{1}=\frac{1 }{1+\frac{1-\text{ρ}_{1}}{\text{S}_{\text{e}}}+\frac{\text{(1}-\text{ρ}_{2})}{\text{S}_{\text{e}}}\frac{\text{(T}-\text{2W)}}{\text{W}}}$ and $\text{k}_{2}=\frac{1 }{1+\frac{\text{ρ}_{1}}{1\text{-S}_{\text{e}}}+\frac{\text{ρ}_{2}}{1\text{-S}_{\text{e}}}\frac{\text{(T}-\text{2W)}}{\text{W}}}$ . In this case, k_1_ and k_2_ are the same in the control and in the intervention groups. Furthermore, when T increases from 2W, k_1_ decreases from $\frac{1 }{1+\frac{1-\text{ρ}_{1}}{\text{S}_{\text{e}}}}$, and k_2_ decreases from $\frac{1 }{1+\frac{\text{ρ}_{1}}{\text{1-S}_{\text{e}}}}$. When S_e_ and ρ_1_ are close to one, these expressions are close to one and zero, respectively.

In order to minimize the corrections for misclassifications, k_1_ should be kept around one and k_2_ around zero. If we choose to use values of k_1_ not lower than 0.50, with a) W varying between six and 15 months, b) T varying from 2W and c) using the corresponding values of S_e_, ρ_1_ and ρ_2_ indicated in Table 1 of the paper, T should be lower than 10 to 11 years. Because the HIV incidence rate to HIV prevalence ratio is 1/T, this ratio should be higher than 0.09 to 0.10 year^-1^. With these values of T, k_1_ varied within the range 0.007 to 0.019.

**B4 Calculations when HIV incidence is not constant**

In this section, we considered that HIV incidence varied linearly with age having an annual slope u, with a value of zero at a_1_. In this case, the probability to be tested recent seroconverter was calculated by considering the history of the individual j and a time-age diagram. We found that this probability could be formulated as $\text{P}_{\text{TR}}=\text{uW}^{2}\text{(A''x}+\text{B''}^{\text{'}}\text{)}$with A'' and B'' given in Table S7.

In the control group, the number of HIV infections for a group of N people uniformly distributed between the ages a_1_ and a_2_ is $\frac{\text{iN(a}_{2}\text{-a}_{1}\text{)}}{2}$. In the control group, the number of HIV infections for a group of N people uniformly distributed between the ages a_1_ and a_2_, and with HIV incidence increasing linearly from zero at age a_1_, with an annual slope u, is$\frac{\text{uN(a}_{2}\text{-a}_{1}\text{)}^{2}}{6}$. We obtained the same value by taking$\text{u}=\frac{\text{3i}}{\text{(a}_{2}\text{-a}_{1})}$.

**Figure legend**

**Figure S1. Time-age diagram for the intervention group.**

**Figure legend:** This figure represents for each individual aged a1 to a2 at time t=0, the periods when the incidence rate was i and the periods (gray areas) when it was xi, x being the effect of the intervention. Birth cohorts are represented by segments having an angle of 45° with the time axis. a, b and c are given in Table S1 as functions of the duration of the intervention.

**Reference**

1. McDougal JS, Parekh BS, Peterson ML, Branson BM, Dobbs T, et al. (2006) Comparison of HIV type 1 incidence observed during longitudinal follow-up with incidence estimated by cross-sectional analysis using the BED capture enzyme immunoassay. AIDS Res Hum Retroviruses 22: 945-952.
